# Chromatin Structure and “DNA Sequence View”: The Role of Satellite DNA in Ectopic Pairing of the *Drosophila* X Polytene Chromosome

**DOI:** 10.3390/ijms22168713

**Published:** 2021-08-13

**Authors:** Aleksandr V. Zhuravlev, Gennadii A. Zakharov, Ekaterina V. Anufrieva, Anna V. Medvedeva, Ekaterina A. Nikitina, Elena V. Savvateeva-Popova

**Affiliations:** 1 Pavlov Institute of Physiology, Russian Academy of Sciences, 199034 Saint Petersburg, Russia; gennadiy.zakharov@gmail.com (G.A.Z.); avmed56@mail.ru (A.V.M.); 21074@mail.ru (E.A.N.); esavvateeva@mail.ru (E.V.S.-P.); 2 EPAM Systems Inc., Saint Petersburg 197110, Russia; 3 Faculty of Biology, Herzen State Pedagogical University of Russia, 191186 Saint Petersburg, Russia; Kate.an21@yandex.ru

**Keywords:** *Drosophila*, polytene chromosomes, *Canton-S*, *agnostic*, ectopic pairing, 1.688 repeats, 372-bp repeats

## Abstract

Chromatin 3D structure plays a crucial role in regulation of gene activity. Previous studies have envisioned spatial contact formations between chromatin domains with different epigenetic properties, protein compositions and transcription activity. This leaves specific DNA sequences that affect chromosome interactions. The *Drosophila melanogaster* polytene chromosomes are involved in non-allelic ectopic pairing. The mutant strain *agn^ts3^*, a *Drosophila* model for Williams–Beuren syndrome, has an increased frequency of ectopic contacts (FEC) compared to the wild-type strain *Canton-S* (*CS*). Ectopic pairing can be mediated by some specific DNA sequences. In this study, using our Homology Segment Analysis software, we estimated the correlation between FEC and frequency of short matching DNA fragments (FMF) for all sections of the X chromosome of *Drosophila* *CS* and *agn^ts3^* strains. With fragment lengths of 50 nucleotides (nt), *CS* showed a specific FEC–FMF correlation for 20% of the sections involved in ectopic contacts. The correlation was unspecific in *agn^ts3^*, which may indicate the alternative epigenetic mechanisms affecting FEC in the mutant strain. Most of the fragments that specifically contributed to FMF were related to 1.688 or 372-bp middle repeats. Thus, middle repetitive DNA may serve as an organizer of ectopic pairing.

## 1. Introduction

Spatial organization of the cell nucleus is an important factor defining the regulation of gene activity, as well as the processes of DNA replication, recombination and reparation. During interphase, chromosomes occupy separate territories in the nucleus, being radially arranged: gene-rich chromosome territories are localized toward the interior, while gene-poor territories are close to periphery [1,2]. In human cells, regions of increased gene expression (ridges) are clustered in spatially distinct gene-enriched domains characterized by irregular forms and low chromatin condensation. These domains are predominantly located toward the nuclear interior. On the contrary, antiridges are relatively gene poor, condensed, transcriptionally inactive and localize closer to the cell envelope. Mechanisms behind the formation and maintenance of such 3D structures are still unclear, possibly due to the interaction between some unknown DNA sequences with nuclear matrix, specific proteins and/or non-coding RNAs [3]. Studying such mechanisms is necessary for understanding the process of gene regulation at the system level. As gene juxtaposition in nuclei facilitates specific chromosomal translocations, 3D chromatin structures can also predict the genetic rearrangements leading to carcinogenesis [4].

Chromosome territories are not rigidly fixed spatial units and show a significant percentage of intermingling, mostly at their borders, that is influenced by gene transcription [5]. Some genes are able to change their location in nuclei, being brought together with the help of actin and myosin motor proteins [6]. Transcription mainly occurs within the nuclear areas enriched in RNA polymerase II (RNAPII), known as transcription factories [7]. The expression level for given genes depends on their proximity to such a factory. The constitutively active genes may nucleate the factory, whereas the others relocalize to it upon their induction [4,7].

In addition to diploid cells, some organisms, such as *Diptera* species, also have polytene cells where chromatids are not segregated after multiple duplications. The giant polytene chromosomes of *Drosophila melanogaster* 3rd instar larvae are characterized by specific banding patterns, which can be revealed by electron and light microscopy. The densely packed thick “black” bands with high DNA content are transcriptionally repressed chromatin areas with low gene density. The largest among them are the intercalary heterochromatin (IH) bands, being late-replicating, under-replicated genomic areas prone to chromosomal breaks, constrictions and non-allelic ectopic contacts formation. *Drosophila* polytene chromosomes harbor about 250 IH sites. The “grey” bands are partially decondensed and more transcriptionally active compared to the IH. Interbands are the most active and the least condensed genomic areas, characterized by the “open” chromatin structure. Bands are united into cytological sections, such as 1A, 1B, 1C … and up to 20F for the X chromosome [8,9,10]. Each type of band contains specific proteins and is enriched for specific genetic elements. Interbands mostly contain the promoters of the constantly active housekeeping genes, being associated with open chromatin proteins such as CHRIZ/Chromator. The “grey“ bands contain multiple active genes but lack CHRIZ, being enriched with RNApol II. The IH bands are composed of tissue-specific genes, being associated with SUUR, D1, lamin B and histone H1 proteins [11]. Notably, such structures are not unique for polytene nuclei, as the chromatin folding and protein composition are conserved in different fly tissues, being closely related to the morphology of the polytene chromosomes. At the same time, the ability to form distant contacts in polytene chromosomes is restricted by their lack of flexibility [12]. Replication timing is similar between polytene and diploid *Drosophila* cells. The late replicating black bands in various tissues correspond to silent chromatin types, with borders enriched for SUUR, lamin and H3K27me3 [12]. This makes polytene nuclei a convenient model to study 3D nuclear organization.

As previously shown by Horchstrasser et al. [13], polytene chromosomes occupy specific spatial domains similar to chromosome territories in diploid nuclei. Chromosomes extend across the nucleus in a Rabl orientation (i.e., their centromeres group near one pole of the nucleus and telomeres near the opposite pole). Chromosomes are coiled in a right-handed fashion, with their 2L and 2R arms being mostly next to each other, as well as the 3L and 3R arms. The loci enriched in IH and ectopic contacts are oriented toward the envelope. However, there are no stable intrachromosomal interactions beyond the distance of two cytological divisions. Thus, chromosomal configuration varies significantly, even though the contacts between the distant loci through the ectopic fibers were not addressed in that study.

Chromosome conformation capture technologies make it possible to investigate nuclear 3D organization with a resolution of the order of one to tens kb. The chromosome conformation capture (3C) method is used to estimate the average frequency of a contact between the two known chromosomal loci in a cell population. Chromosome conformation capture-on-chip (4C) technology allows spatial contacts of a selected genomic site to be assayed with all unknown distant sites. In the chromosome conformation capture carbon copy (5C) method, a massive analysis of contacts between specific loci across the entire genome is performed. The Hi-C method reveals spatial contacts at the level of the whole genome [14]. The complex net of such physical contacts uncovers the chromosome topology in detail, assaying interactions between genes and their regulatory elements.

Using Hi-C technology, the spatial structure of the *Drosophila* genome was studied for both diploid and polytene nuclei with a resolution of 15 kb [15]. Polytene bands nearly correspond to topologically associated domains (TADs) with a mean size of 195 kb. They are conserved for polytene and diploid nuclei. TADs are persistent throughout fly development, and are formed by the axial condensation of the chromatin fiber. The putative role of a TAD is DNA compaction rather than regulation of gene activity. Stable interactions between the different TADs were not observed, with is consistent with the variable long-range chromosomal conformation in the nucleus [13]. In the other study on *Drosophila* embryonic nuclei, the long-range interactions between Polycomb-repressed domains were found. The hierarchically organized domains were associated with active and repressive epigenetic modifications of chromatin [16]. Ectopic pairing was not considered in Hi-C studies, as its frequency was low and such long-range contacts were beyond the limit of the resolution. Thus, light and electron microscopy remain the most appropriate methods by which to study the ectopic pairing.

Ectopic contact is morphologically observed as an intimate association of IH bands or as an unstructured fiber connecting two IH bands. As the paired chromosome sections seem to be covalently linked due to the chromatids recombination/reparation, ectopic pairing is not disrupted upon squashing with acetic fixation [12,17]. The method of squashed preparations was used to estimate frequency of ectopic contacts (FEC) in several *Drosophila* strains. FEC can be expressed as the total number of ectopic contacts between a given section pair.

*agn^ts3^* is a *D. melanogaster* mutant with a dysfunction of LIM kinase 1 (LIMK1), the main regulator of actin polymerization in nervous cells. This mutant strain shows multiple cognitive impairments, being the model object for Williams–Beuren syndrome [18]. a/t-rich *agnostic* locus (X:11AB) is predisposed to mutations. Its length varies, probably due to spontaneous unequal recombination [19]. Impairment of LIMK1 and actin dynamics affects the spatial organization of chromatin [20]. For *agn^ts3^*, as well as the wild-type strains *Canton-S (CS)*, *Berlin* and *Oregon-R*, multiple polymorphisms were found in the *limk1* gene and flanking sequences. In *agn^ts3^*, there is also mobile S-element insertion downstream *limk1* [21,22]. The *agn^ts3^* FE*C*s are significantly higher compared to the wild-type strains [22]. Though profiles of ectopic pairing have shown a significant inter-strain variation, the pairing often occurs at the same loci. Thus, DNA sequence itself may define which loci can form a contact, whereas the epigenetic factors and/or activity of specific genes, such as *limk1*, affect FEC values by changing chromatin properties and tendency to pair.

There are several models of ectopic contact formation with the IH bands: (1) Pairing of “sticky ends” of the short repeated sequences within the areas of the DNA breaks that occur due to under-replication. (2) Pairing between the extended homologous DNA sequences. Generally, ectopic pairing occurs between the areas of chromosomes that do not show a significant homology, though it has been observed for some bands. (3) DNA branch migration upon replication mistakes, presumably due to the restricted homology between the associating sequences. (4) Pairing mediated by specific heterochromatin-associated proteins [9]. At least in the first three cases, presence of identical DNA sequences is crucial for pairing according to the principle of complementarity. In other words, high FECs should correlate with high frequencies of matching short DNA fragments (FMF) for the contacting regions.

The method of squashed preparations is imprecise, as it permits the localization of areas of contacts with a resolution of tens to hundreds of thousands bp. Hence, it gives no information about the specific DNA sequences involved in ectopic pairing. The bioinformatics approach helps to handle this problem. To estimate correlations between Drosophila FEC and FMF, we designed software called Homology Segment Analysis [23]. The current version of the software performs the following:

1. DNA sequences of the chromosome sections (A) are taken one by one, searching for short single-stranded fragments (k-mers) of a given length that are the same as fragments of the other sections (B). For each pair of sections (A-B), FMF is calculated as the total number of matching fragments for both DNA chains. To increase matching specificity, short DNA repeats (microsatellites) can be excluded at this stage.
2. For each section A, the rho value of the Spearman correlation between FEC and FMF is computed. Both specific and unspecific correlations are considered (FEC and FMF values correspond to the same or different section pairs, respectively).
3. For all sections A, the average rho values (R) and the proportion of statistically significant FEC-FMF correlations (P) are calculated at different fragment lengths, for different Drosophila strains, and statistically analyzed.
4. For each A-B pair, the list of the matching fragments is generated and ordered according to their numbers of occurrence. This lets us reveal short DNA sequences that specifically impact FEC-FMF correlation.

Steps 1–3 can be performed for sections within chromosome parts of different sizes.

Using a previous version of the software, we showed a positive correlation between *Berlin*/*agn^ts3^* FEC and FMF for identical short (30–50 nt) DNA fragments. In that research, we specifically focused on the X:11AB region and its contacts with the other sections of the X chromosome. Most of the fragments found to putatively make impact into ectopic pairing were similar to the middle repetitive DNA 372-bp sequence and the 1.688 g/mL satellite DNA family [24]. The distribution and properties of 372-bp indicate its possible role in *Drosophila* dosage compensation and primary sex determination [25]. The 1.688 satellite DNA is abundant in *Drosophila* genome (2%), being localized both in heterochromatin and in euchromatin domains (1860 and 168 copies, respectively), mainly on the X chromosome. This satellite family includes 360/359-bp, 353-bp and 257-bp subfamilies [26]. The 1.688 satellite DNA is known to produce small RNAs that participate in the localization of male-specific lethal complex (MSL) on the X chromosome, increasing male survival [27]. The above point to a striking connection between the ectopic pairing and non-coding RNA-dependent processes of dosage compensation. 359-bp also produces a long non-coding RNA that interacts with centromeres of all major chromosomes, participating in their mitotic segregation [28].

In this study, we calculated FEC-FMF correlations for all pairs of the X chromosome sections (1A–20F) in *CS* and *agn^ts3^* strains at fragment lengths of 10–60 nt. Each section begins with an IH band, hence all of them are theoretically able to participate in ectopic pairing. The effect of the proximity of chromosome sections on FEC-FMF correlation value was estimated by analyzing the average correlation values within the chromosome parts of different sizes. For fragments specifically making the contribution to FMF, the biological nature was determined using NCBI Blast software. Most of them showed a high percentage of identity with 1.688 and 372-bp repeats, as well as related genes. These repeated sequences were either concentrated in genomic regions predisposed to ectopic pairing or governing pairing themselves, via DNA-DNA complementary binding or indirectly with the help of some unknown protein or RNA factors.

## 2. Results

### 2.1. FEC-FMF Correlations for the Whole X Chromosome

**Figure 1:**
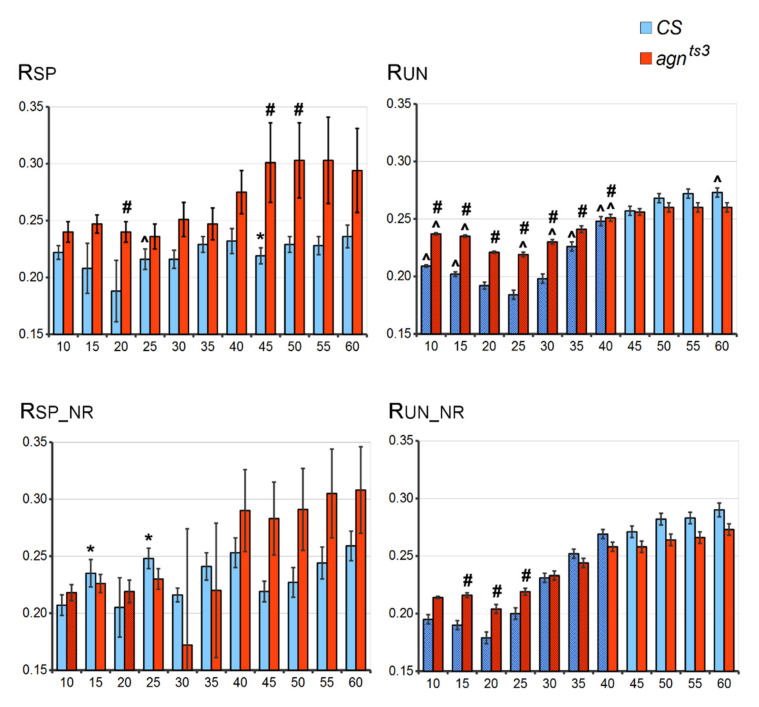
R values at different fragment lengths. *X* axis: L (nt). *Y* axis: R (conventional units). Difference: # from *CS*, ^ from the case with excluded repeats, * from the case with unspecific correlation, shading – difference from R calculated at L50 (two-sided Mann–Whitney U-test; *p* < 0.05). Standard error of mean is shown. Here and below: SP—specific, SP_NR—specific with repeats exclusion, UN—unspecific, UN_NR—unspecific with repeats exclusion. Sampling number: for specific correlations, *n* = 6–20 (*CS*), 5–28 (*agn^ts3^*); for unspecific correlations, *n* = 568–2235 (*CS*), 667–2987 (*agn^ts3^*). Total number of R estimations: for specific correlations, *n* = 90 (*CS*), 95 (*agn^ts3^*); for unspecific correlations, *n* = 10,620 (*CS*), 11,210 (*agn^ts3^*).

The average values of statistically significant FEC-FMF Spearman rho correlations (R) were calculated for all the pairs of sections of the X chromosome (Figure 1). R specific (R_SP_) varied within 0.18–0.3, corresponding to a rather weak positive FEC-FMF correlation [29]. R_SP_ was slightly higher for *agn^ts3^* compared to *CS*, especially at a fragment length (L) of 45–50 nt (L45-50), possibly due to a larger FEC in *agn^ts3^*. Repeats exclusion did not significantly affect R_SP_, except for its small decrease at L25 in *CS*. For both *CS* and *agn^ts3^* strains, there were no significant R_SP_ differences from those calculated at L50.

To check the correlation specificity, R unspecific (R_UN_) was calculated as the average value of statistically significant rho correlations for all the different section pairs A and B. Such a shuffle of sections permits estimation of the “false“ correlation between the inappropriate FEC and FMF values. R_UN_ was nearly the same as R_SP_; thus, if rho correlation appears to be statistically significant by chance, its average value does not differ from that of the “true” correlation. R_UN_ was higher for *agn^ts3^* compared to *CS* at L10–40; however, there were no interstrain difference at a larger L. For both strains, R_UN_ grew with the length of fragments, reaching maximum values at L50–55. The exclusion of short repeats reduced R_UN_ values for small Ls (10–20). Thus, microsatellites seem to have a significant impact on unspecific correlations. At a larger L (45–60), repeats exclusion did not significantly affect R_UN_. For both strains, it was mostly impossible to distinguish between R_SP_ and R_UN_. Thus, the average rho value rather weakly reflected the probability of ectopic pairing.

**Figure 2:**
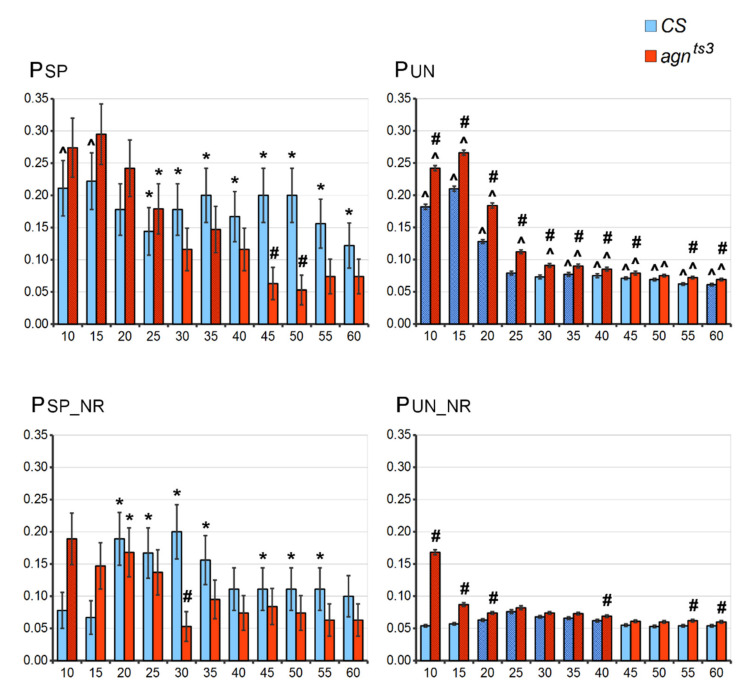
P values at different fragment lengths. *X* axis: L (nt). *Y* axis: P (conventional units). Difference: # from *CS*, ^ from the case with excluded repeats, * from the case with unspecific correlation, shading-difference from P calculated at L50 (Chi-square test; *p* < 0.05). Standard error of sample proportion is shown.

The picture was different for the proportions (P) of statistically significant correlations (their share of all correlations). P specific (P_SP_) showed a non-linear variation along with L growth (Figure 2). For *agn^ts3^*, the first P_SP_ maximum (about 0.3) was observable at L15 (i.e., 30% of all sections forming the ectopic contacts had a significant FEC-FMF correlation); then, P_SP_ dropped to 0.12 at L30, returning to about 0.15 at L35. Finally, P_SP_ dropped to 0.05 at L50, being nearly equal to the probability of finding an FEC-FMF correlation by chance (*p* < 0.05). This corresponds to the virtually complete absence of section-to-section matching for fragments longer than 60 nt (FMF was zero for the most sections). For *CS*, the whole picture was the same, but P_SP_ changes with L were not significant. Most importantly, we observed a striking interstrain P_SP_ difference at L45–50: P_SP_ value remained rather high for *CS* but not for *agn^ts3^*. The fragment length of 50 nt has been previously shown as optimal to detect FEC-FMF correlations for the other *Drosophila* wild-type strain, *Berlin* [24]. Thus, at least in some strains, high FMF (L50) can serve as a predictor of ectopic pairing with a probability of about 20%.

P unspecific (P_UN_) values showed a decrease along with the L increase, down to 0.05–0.07, which was close the theoretically expected *p* value of 0.05. For *CS*, P_SP_ is significantly higher than P_UN_ at L25–60. For *agn^ts3^*, there were no significant differences between P_SP_ and P_UN_, except at L25. Hence, *agn^ts3^* strain clearly demonstrated less FEC-FMF correlation specificity compared to the wild-type strain. Repeats exclusion significantly decreased P_UN_. This also proves that short simple repeats abundant in *Drosophila* genome make a significant impact into unspecific FMF-FEC correlations. The exclusion of microsatellites decreased the interstrain P_SP_ difference, though P_SP_ remained significantly higher for *CS* at L30. Putatively, short repeats are parts of some longer DNA fragments that specifically have an impact on FEC-FMF correlation, therefore repeat filtration can simultaneously remove these fragments from samplings used to calculate FMF.

The observable P-L dependence seems to be the summary effect of the following trends: (1) Both P_SP_ and P_UN_ are high for short fragments that are randomly distributed within genome. (2) Both P_SP_ and P_UN_ are low for long fragments, but P_UN_ drops more rapidly along with L increase. Exclusion of short repeats lowers the probability of FEC-FMF correlation, so we do not recommend it be performed for large Ls. High FMF calculated at L50 seems to indicate the higher probability of specific ectopic pairing for *CS* X chromosome sections. However, the share of statistically significant correlations remains rather low (about 20%). Thus, ectopic pairing is mostly governed by some other factors not taken into account in the FMF calculated according to the above scheme.

### 2.2. FEC-FMF Correlation for Chromosomal Regions of the Different Length

Ectopic contacts are mainly formed between sections that are not too far from each other (i.e., are separated by not more than 10–20 sections) [30]. To check the influence of the intersection distance (D) on R and P values, we performed FEC-FMF calculations for sections within chromosome zones of specific length D (for a more detailed description, see Section 4.2, Stage 9).

**Figure 3:**
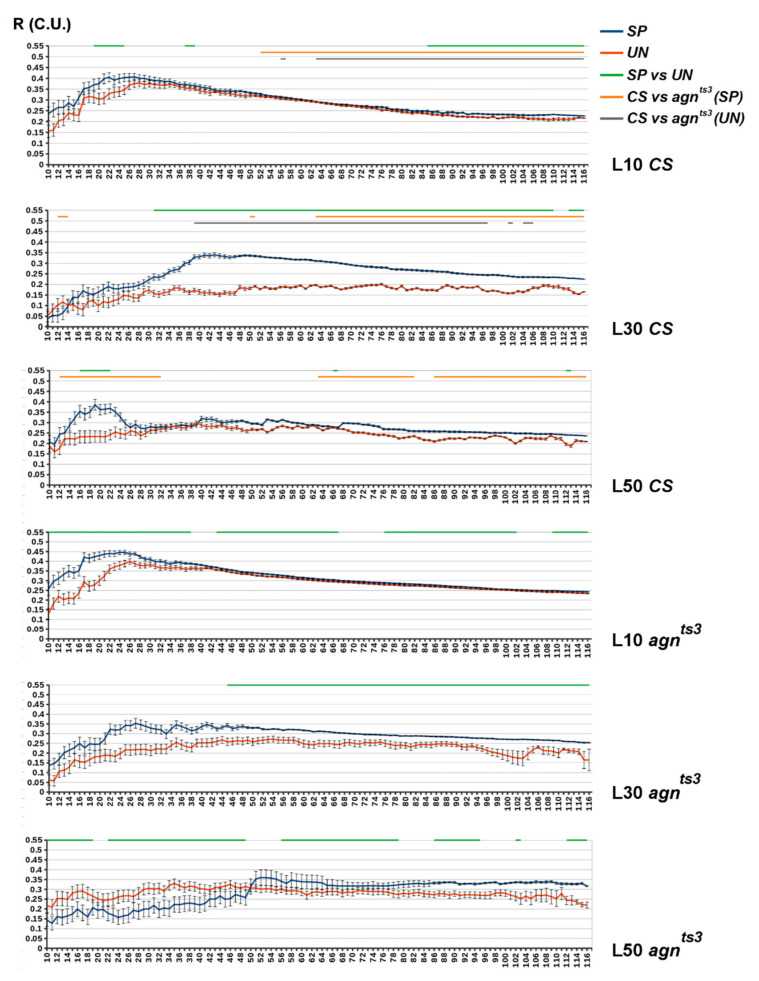
The average R values for the chromosomal zones of different intersection distances (D). *X* axis: D—zone length (in sections). *Y* axis: R (conventional units). Standard error of mean is shown. The areas of D with statistical differences are shown by straight lines above the diagram: green—SP vs. UN, orange—SP (*CS*) vs. SP (*agn^ts3^*), grey—UN (*CS*) vs. UN (*agn^ts3^*) (two-sided Mann–Whitney U-test; *p* < 0.05, *n* = 120–D).

The average R values for different D are shown in Figure 3. In *CS*, R_SP_–R_UN_ difference (R_DIFF_) was about 0.05–0.1 for small fragments (L10) and small D (10–25). However, in most cases it was insignificant because of the high R variance. For larger D, R_DIFF_ was small but statistically significant, save for the highest D values obtained for very small samplings. The highest R_DIFF_ could be observed at L30, with its maximum at D40–45. At L50, there was no significant *CS* R_DIFF_, save for a few D values. The picture was similar for *agn^ts3^*, but its R_DIFF_ was smaller at L30 and even became negative at L50 (D < 50). The interstrain difference was small in most cases, but at L30 *CS* had apparently lower R_UN_ compared to the mutant strain. Generally, the highest R_SP_ and R_DIFF_ could be observed for rather small Ds (up to 40), confirming the hypothesis that ectopic contacts mainly occur between spatially close bands.

The exclusion of microsatellites affected the *CS* R_DIFF_, which approached zero at L30 and increased at L50 with most D values (Supplementary Materials, Figure S1). R_DIFF_ (L50) remained maximal for small D. Hence, the highest FEC-FMF positive correlation can be observed within the short areas of the X chromosome. In *agn^ts3^*, the area of D with positive R_DIFF_ shrinked at L10 and increased at L50, probably due to the increase in correlation specificity after repeats filtration. However, the range of D with the negative R_DIFF_ increased at L30. Hence, *agn^ts3^* shows pronounced negative FEC-FMF correlations for relatively small Ds.

**Figure 4:**
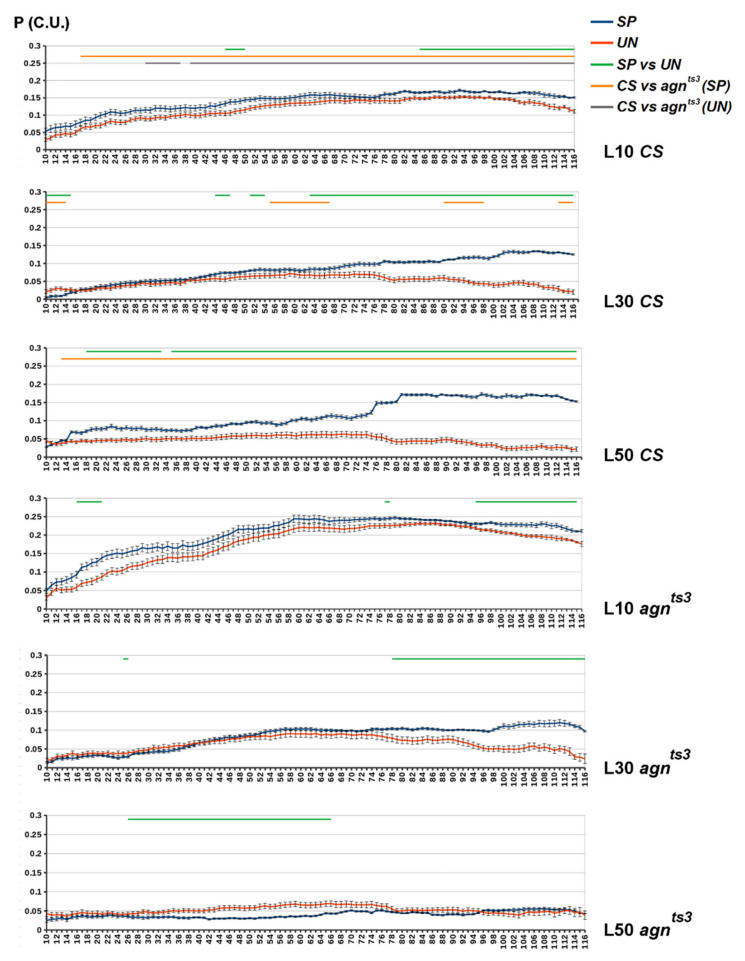
The average P values for the chromosomal zones of different intersection distances (D). *X* axis: D—zone length (in sections). *Y* axis: P (conventional units). Standard error of mean is shown. The areas of D with statistical differences are shown by straight lines above the diagram: green—SP vs. UN, orange—SP (*CS*) vs. SP (*agn^ts3^*), grey—UN (*CS*) vs. UN (*agn^ts3^*) (two-sided Mann–Whitney U-test; *p* < 0.05, *n* = 120–D).

For the *CS* P values, we can see an obvious increase in P_SP_–P_UN_ difference (P_DIFF_) along with increases in D and L values (Figure 4). At L50, occurrence of specific correlations became significantly higher for almost the whole range of D. The highest values of both P_SP_ and P_UN_ could be observed in *agn^ts3^* at L10, but P_DIFF_ was relatively small. There was also an increase in *agn^ts3^* P_DIFF_ along with D growth at L30, but it completely vanished at L50 and even became negative for the mean D values. At L50, the interstrain P_SP_ difference was maximum and P_UN_ was equal for both strains, being close to the theoretically predicted value of 0.05. This confirms results obtained for the X chromosome without division onto D zones. Thus, the most specific FEC-FMF correlation for the whole X chromosome can be observed in *CS* at L50.

The exclusion of microsatellites did not generally change the picture for *CS* P_DIFF_, the value and significance of which increased along with the growth of D and L (Figure S2). P_UN_ was about 0.05, similar to that for the whole X chromosome (see Figure 2). P_SP_ (L50) was somewhat lower compared to the case without repeats exclusion, probably because of simultaneous filtration of long fragments specifically making impact into FMF. For *agn^ts3^*, P_DIFF_ was mostly negative at L30. This may indicate some inverse relationship between FEC and FMF for that strain. At L50, the mutant P_DIFF_ was negative for D < 30 and positive for D > 60, though in both cases its values were small. Thus, in contrast to *CS*, *agn^ts3^* does not show a stable increase in P_DIFF_ along with D and L growth.

### 2.3. Sections Prone to Ectopic Pairing

Some chromosomal areas are known to be prone to ectopic pairing. The total FEC number (FEC_TOT_) is a number of ectopic contacts between a given section and all the other sections of the X chromosome. There was a strong positive FEC_TOT_ correlation for *CS* and *agn^ts3^*: rho = 0.757 (*p* < 0.001, *n* = 119). After replacing the exact FEC with the indices of presence (1) or absence (0) of contacts, the interstrain correlation decreased but remained significant: rho = 0.540 (*p* < 0.001). Thus, in both strains, ectopic contacts are mainly formed by the same sections, and sections with a high FEC are usually localized at the same position (see also [30]).

According to [31], there are five chromatin features (F) increasing the probability of the *Drosophila* X chromosome ectopic pairing, such as *Dm225* and *Dm234b* genes hybridization sites, ectopic conjugation, weak points, late replication and giant palindromes. For each F, we assigned the value of 1 or 0 to sections depending on whether they had or did not have a specific F. Then we calculated the F index (Ind) as the sum of values for each section (F_SUM_) divided by 10. As expected, there was a positive correlation between FEC_TOT_ and F_SUM_: rho (*CS*) = 0.357 (*p* < 0.001); rho (*agn^ts3^*) = 0.252 (*p* < 0.01). Replacing the exact values with indices of presence (1) or absence (0) made correlation insignificant.

**Figure 5:**
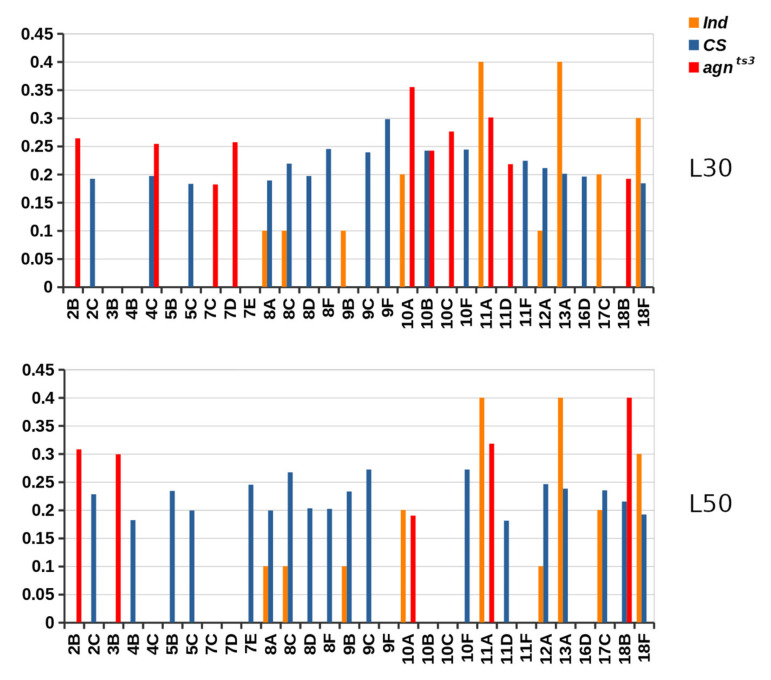
X chromosome sections showing statistically significant FEC-FMF correlations. Ind—the index of chromatin features (F). Y value: R_CORR_ (for *CS* and *agn^ts3^*) and Ind values (C.U.).

At the same time, we did not observe any correlation between FEC_TOT_ and the total FMF number for each section (FMF_TOT_) calculated at L30 or L50. FEC-FMF correlation was specifically observed only for a set of the X chromosome sections (Figure 5). Three of them (10B, 11D and 18B) coincided for both *Drosophila* strains. Only the minor part of sections (26.1% for *CS*, and 18.2% for *agn^ts3^*) had chromatin features predisposing them to ectopic pairing. Considering a rather moderate FEC_TOT_-F_SUM_ correlation, we state that the indicated specific chromatin properties can facilitate ectopic pairing, but are not necessary for that. For most sections, ectopic pairing seems to be governed by some other mechanisms possibly related to specific DNA sequences within the interacting areas.

### 2.4. The Biological Nature of Sequences Making Impact into Ectopic Pairing

To reveal the molecular nature of the fragments most contributing to FMF, we analyzed their sequences using NCBI Blast. The first fifty L30 and L50 fragments with the maximum number of occurrences (NOs) were assayed, both for all sections (set I) and for a set of sections showing *CS*- or *agn^ts3^*-specific FEC-FMF correlations (sets II and III, respectively). To simplify the analysis, we did not consider fragments with NOs ≤ 10 and considered only one of the fragments with the equal NO values.

**Figure 6:**
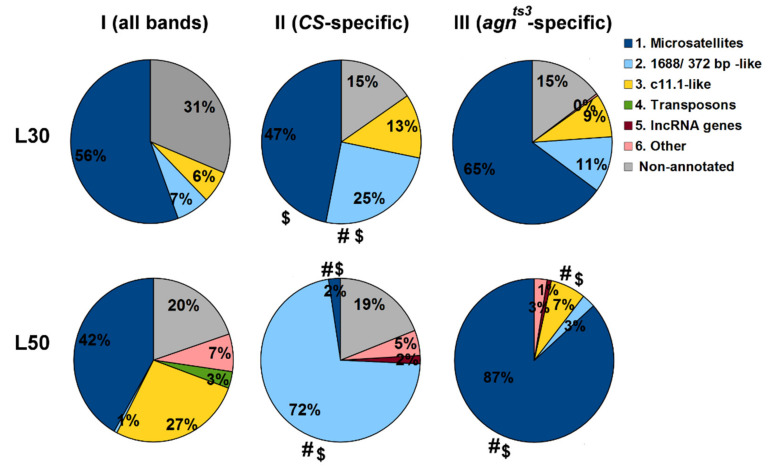
Short DNA fragments that contribute to FMF. Difference: # from I, non-annotated sequences included when calculating the proportions; $ from I, non-annotated sequences excluded (Chi-square test of two proportions; *p* < 0.05, *n* > 200).

BLAST analysis made it possible to divide all matching fragments into six classes: (1) Microsatellites, such as a_n_, t_n_, (at)_n_, (aat)_n_, (gata)_n_, (agata)_n_, (tcccag)_n_ and so on, the maximum motif length being of six. (2) Fragments showing a high percentage identity (90–100%) with 1.688 and 372-bp repeats, as well as with some genes such as *c11.1*, *kl22 Drak* intron and related. (3) Fragments showing a high percentage identity with sequences of *c11.1* and *kl22 Drak* but not with 1.688 or 372-bp repeats. (4) Fragments of transposon HB1. (5) Fragments of long non-coding RNA genes and retrotransposon roo-900. (6) Other; mostly the sequences with an unknown molecular nature, or those for which BLAST returned no result. Up to 31% of fragments (with NOs < 50) remained non-annotated. The results are present in Figure 6. The full list of fragments, along with their NO, molecular nature and BLAST sequence identity, is shown in Table S1.

In set I, microsatellites constituted the major class of fragments. The above is not surprising, as the program searches fragments one by one, with a step of one nucleotide, generating a large number of identical short sequences from an extended area of repeats. At the same time, the simplest repeats, such as (at)_n_ and (ta)_n_, prevailed at L30, but not at L50. The microsatellite (tcccag)_n_, appeared to have a maximum NO at L50 and a third-rank NO at L30, indicating its abundance in *Drosophila* genome. The NO was also high for fragments belonging to class 3, while class 2 was nearly absent at L50. Transposon parts constituted at least 3% of L50 fragments.

In set II, containing fragments of sections with *CS*-specific FEC-FMF correlations, NOs for microsatellites significantly decreased, especially at L50. At the same time, NOs for 1.688/372-bp-related sequences increased many times (up to 72% at L50). P_SP_ was at its maximum for *CS* at L50 (see Figure 2 and Figure 4). This clearly indicates that microsatellites mostly contribute to unspecific FMF, and 1.688/372-bp-related sequences mostly contribute to FEC-FMF specific correlation. The latter is in agreement with our previous data obtained for *Berlin* [24]. (at)_n_ was the only microsatellite that seemed to have an impact on *CS*-specific correlation. The fact that (at)_n_ and complementary sequences constituted 66% of microsatellites and 31% of all *CS*-specific fragments at L30 explains why the NO was high for *CS*-specific sets of selected microsatellite fragments. Sequences of class 3 also contributed to FEC-FMF correlation at L30.

On the contrary, for *agn^ts3^*-specific fragments (set III) we observed an increase in microsatellite NOs, which reached their maximum (at)_n_ at L30 and (gata)_n_ at L50. For classes 2 and 3, the NOs insignificantly grew at L30, although for class 3 the NO dropped at L50. In *agn^ts3^*, P_SP_ was only slightly higher than P_UN_ at L30, with no difference at L50 (see Figure 2 and Figure 4). The positions of sections with specific FEC-FMF correlations were different for *CS* and *agn^ts3^* (Figure 5). Taken together, our data show that 1.688/372-bp-related sequences are associated with ectopic pairing in *CS*, with either a minor or no association for *agn^ts3^*.

## 3. Discussion

Ectopic pairing is a long-range interaction that occurs with a relatively low frequency between the IH bands of the *Drosophila* polytene chromosomes. Though ectopic contacts can be easily observed using light microscopy, their molecular nature and functional role in the nuclear 3D organization remain obscure. The *agn^ts3^* strain was shown to have a significantly higher FEC compared to the wild-type fly strains. At the same time, some IH bands are more prone to form ectopic contacts compared to the others. In *Drosophila* reciprocal hybrids of *Berlin* and *agn^ts3^*, specific bands of the X chromosome demonstrate either matroclinic or patroclinic inheritance of high FEC values [24]. Thus, ectopic pairing obviously has both genetic and epigenetic basis.

As molecular processes of ectopic pairing are not studied in detail, we cannot say a priori how long a zone of local DNA pairing should be to initiate a contact formation. Neither can we say whether g/c rich sequences will interact preferably over a/t rich due to the higher binding energy or if the tendency will be reversed, as a/t regions are often nucleosome free, easy to melt, frequent in genome, and therefore may be more prone to misalign. In our study, we have made a simple assumption that all cases of local DNA match will increase the probability of the ectopic contact formation. The optimal length of the areas should be the tread off the specificity of matching and the probability to find a relatively long specific area in genome. We have revealed the optimal fragment length to be about 50 nt. The ability to consider cases of incomplete matching would let us work with longer fragments, but the current version of Homology Segment Analysis software does not permit us to work with local mismatches. FMF values do not depend on the relative orientation of fragments (e.g., they are the same for the sequential parts of long (at)_n_ repeats and several short non-overlapping (at)_n_ repeats). The fragments of all chromosome sections were considered, though only the IH bands participate in ectopic pairing. We believe that the above has a rather small effect on FMF, as the IH bands are densely packed, containing much more DNA than interbands. FEC was zero for most of the section pairs, and the range of FEC variation was rather narrow: in most cases, the FEC equaled 1 or 2 in its Spearman rank correlation. In addition to DNA sequence, epigenetic factors greatly influence FEC values.

As a result, we obtained a rather moderate average FEC-FMF Spearman correlation rho value (R of 0.3), as well as the proportion of section pairs for which the correlation was significant (P of 0.2). Nevertheless, for *CS*, P appeared to be higher than that of unspecific correlations, both for the whole X chromosome and for almost the entire range of the lengths of its parts. Moreover, we found that not all DNA sequences, but mainly those related to 1.688 or 372-bp repeats, contribute to FEC-FMF correlations. This cannot be explained by the predominance of such sequences in the *Drosophila* X chromosome, as, according to our data, the most common matching repeats were (at)_n_ at L30 and (tcccag)_n_ at L50 (Table S1). The other microsatellites also contributed to FMF, but only (at)_n_ seemed to make an impact on the *CS*-specific FEC-FMF correlation. In *agn^ts3^*, FEC-FMF correlations are generally unspecific, despite the higher FEC for this strain. Similarly, the X—X:11AB FEC-FMF correlation was lower in *agn^ts3^* compared to *Berlin* [24]. At L30, specific correlations still could be observed for about 10% of bands participating in *agn^ts3^* ectopic pairing. Microsatellites mostly contributed to *agn^ts3^*-specific sets of matching fragments.

Satellites are multi-copy tandem DNA repeats classified according to the length of their monomers: microsatellites (1–10 bp), minisatellites (10–100 bp) and satellites (>100 bp). *Drosophila* satellites are involved in multiple cell processes, such as dosage compensation, heterochromatin establishment, gene activity regulation, maintenance of genomic architecture, chromosome segregation and development [32]. Satellite DNA is one of the most abundant and fast evolving components of genome, being the major part of the constitutive heterochromatin in eukaryotes. There are at least 14 families of highly repeated *D. melanogaster* satellite DNA [33]. The most abundant satellite repeats of the X chromosome are aatat, aagag, 359/372/260-bp, and IGS [34].

The 372-bp satellite is a conservative a/t rich *Drosophila* repeat concentrated on the euchromatin of the X chromosome, located mainly between cytogenetic regions 4 and 15, with about 300–400 copies per haploid genome. Its distribution and sequence features suggest its participation in the primary fly sex determination and dosage compensation [25]. This satellite DNA is homologous to the 1.688 g/mL class of satellites predominantly localized to the centromere heterochromatin of the X chromosome [34,35]. The 1.688-3F satellite expresses a siRNA-generating hairpin dsRNA that increases males survival, regulating the male-specific lethal complex (MSL) positioning on the X chromosome. It is possible that siRNA affects dosage compensation by modifying chromatin at the 1.688 repeat [27]. It should be noted that the *agnostic* locus in *agn^ts3^* does not show dosage compensation [36,37]. Along with a lack of FEC-FMF correlation, this may indicate some deregulation in 1.688 or 327-bp activity in *agn^ts3^*. The 1.688 sequence shows a significant intraspecific nucleotide divergence: 10% for heterochromatin and 27% for euchromatin. Hence, the chromatin structure seems to influence the rate of 1.688 evolution [26]. In our study, the identity between the matching fragments with strain-specific FEC-FRF correlations and 1.688 satellites from 3C and 10Ep bands [35] was about 100% at L30 and slightly below 100% at L50.

Polytene chromosomes normally have low flexibility, extending across the nucleus in a Rabl orientation, with no stable interaction between loci 1-2 cytological divisions apart [13]. The dysfunction of the two interband-associated proteins, Chromator and JIL-1 kinase, leads to dramatic impairment of polytene chromosome morphology (i.e., band misalignment, curling and numerous ectopic contact formations) [38]. As the pattern of ectopic contacts is determined early in development, it should rather reflect the morphology of diploid embryonic nuclei with long-range interactions (e.g., between Polycomb-repressed domains) [16]. Thus, the functional role of ectopic pairing in chromatin spatial organization is questionable. At the same time, it seems to represent some aspects of nuclear 3D structures typical of the early stages of fly development.

The molecular processes that govern ectopic pairing are still poorly understood. In the *Drosophila* interphase nucleus, blocks of heterochromatin tend to associate with each other. The association does not require similar sequences such as (aagag)_n_ satellite to be located within the contacting areas. Presumably, their associations are mediated by proteins that recognize the general features of heterochromatin, such as specific histone modifications, repetitiveness, late replication and low activity [39]. Nevertheless, the restricted homology at the IH areas containing DNA breaks may be important for ectopic contact formations. Probably, it results from the joining and ligation of truncated DNA ends between the IH bands. The level of suppressor of under-replication *(SuUR)* gene expression positively correlates with FEC. *SuUR* overexpression enhances the IH under-replication and ectopic pairing only before the third instar larval stage [17].

In accordance with the above, FEC as a phenotypic trait is determined at the embryonic stage: the high temperature (37 °C) applied at that stage increases FEC in *CS* without an effect in *agn^ts3^*. This seems to be connected to strain-specific properties of heterochromatin that begins to form at this stage [22]. High FEC values in *agn^ts3^* may reflect the increase in strain-specific recombination/reparation activity, as well as the activity of some chromatin proteins, such as the Polycomb group, HP1, Chromator and JIL-1. It is interesting to note that both JIL and *agn^ts3^* LIMK1 genes bring the insertion of a mobile S-element that may theoretically cause some interaction between these genes [21,40]. *agn^ts3^* is also characterized by significant changes in miRNA expression profile compared to the wild-type strains *CS* and *Berlin* [21,24]. The mutant-specific heterochromatin properties may affect the wide profile expressions of genes, including non-coding RNA genes.

On the contrary, miRNAs may affect the expression of heterochromatin proteins and the tendency of the IH bands to form ectopic contacts. The targets of some of miRNAs participating in the development of human neurodegenerative disorders are Swi/Snf-like chromatin remodeling complex, REST factor that recruits histone deacetylases (HDAC), and SIRT1, a NAD^+^-dependent HDAC involved in heterochromatin formation. miR-124 and miR-34c negatively regulate HDAC1/2 and SIRT1, respectively [41]. *Drosophila* miR-124 and miR-34 are decreased in *agn^ts3^* compared to *CS* [21]. Hence, the high percentage of heterochromatin in *agn^ts3^* may be due to the increase in HDAC activity.

*agn^ts3^* is shown to impair the activity of LIMK1, the main regulator of actin remodeling [18]. The filamentous and globular actin differently affect chromatin conformation, as well the activity of HDAC [42]. Actin is widely involved in the regulation of genetic apparatus, including transcription machinery [20]. Active forgetting also depends on an LIMK1-dependent signaling cascade [43]. This reveals a possible connection between the molecular processes of heterochromatin formation in the early embryogenesis of fruit flies and the *Drosophila* ability to learn, memorize and forget.

Heterochromatin proteins such as the Polycomb group and HP1 may influence ectopic pairing by bringing the IH bands closer together in space or linking them to the envelope [17]. HP1 promotes the formation of chromosome loops, facilitating the coalescence of dispersed middle repeats such as micropia retrotransposon and non-coding RNA gene αγ [44]. H3K9me3 modification of 1.688 satellite creates a binding site for HP1 [45,46]. Hence, FEC-FMF correlation can be theoretically explained by HP1-dependent juxtaposition of the 1.688-containing under-replicated IP bands, followed by ligation of the double-stranded DNA ends. A similar role of 1.688-3C in ectopic pairing of the spermatocyte X chromosome was proposed in [34,47]. Previously, 1.688 was proposed to influence chromatin architecture by interacting with proteins of the nuclear matrix, such as Topoisomerase II and satellite binding protein. The 1.688 satellite may also participate in long-range interactions regulated by siRNA-dependent chromatin modifications [48]. Though 372-bp repeats are localized to euchromatin, they may also play a role in bringing together chromosome sections that form ectopic contacts.

In summary, our computational data confirm the hypothesis that *Drosophila* satellite DNA such as 1.688 and related sequences, can participate in long-range interactions between the IH bands. The lower FEC-FMF correlation for *agn^ts3^* relative to *CS* may indicate less specificity of ectopic pairing in the mutant strain, similar to Chromator/JIL-1 mutants. Some proteins or non-coding RNAs, possibly produced by 1.688-like repeats, can mediate the interaction. Alternatively, they can affect heterochromatin properties and/or DNA replication within IH bands, making them more prone to pairing. Further studies are necessary to prove this experimentally.

## 4. Materials and Methods

### 4.1. FEC Matrices

FEC matrices for the X chromosome sections of *CS* and *agn^ts3^* strains were taken from [30]. Files containing FE*Cs* for all X chromosome sections of both strains were supplied with Homology Segment Analysis software. Each matrix was built on the data obtained by orcein staining of squashed preparations of *Drosophila* 3rd instar larvae. For each strain, 30 larvae were taken, and about 20 nuclei were examined for each larva. FE*Cs* were calculated as the total number of ectopic contacts between the given section pair. The example of an ectopic contact is shown in Figure S3.

### 4.2. Bioinformatics Analysis

FEC-FMF correlations were estimated using Homology Segment Analysis software (Zhuravlev A.V., Zakharov G.A.; Pavlov Institute of Physiology, Saint Petersburg, Russia). The program is written on Python3. The calculations in the paper were performed using the latest version of the program, freely available at [23]. For both strains, we used the *D. melanogaster* X chromosome sequence genome assembly Release 6 (dm6) [49]. As there are no full genomic sequences for these strains, and S-elements are relatively short and can be found at various positions in different *Drosophila* strains, we do not consider it justified to include *limk1* S-element in *agn^ts3^* genome sequence to compute its FMF. All interstrain differences are expressed here as FEC differences. The borders of the X sections were chosen according to Flybase data (www.flybase.org; accessed on 20 February 2021): 1A–20F, except 20B missing some nucleotides, totally 119 sections.

To install and run all the scripts, see the Readme file in the main directory.

The computational algorithm is as follows:

1. Sequential selection (with a step of 1 nt) of short DNA fragments of a given length (L) from one specific section of the X chromosome.
2. Search of the section fragments, as well as the complimentary fragments, within all the other sections of the X chromosome, using Aho-Corasick algorithm.
3. For each X chromosome section (B) except A: calculation of normalized frequency of all fragments matching for A and B (FMF(A-B)). The average FMF for all chunks of 10 kb length is equal to 1. The list of the localized fragments is saved for each B.
4. Stages 1–3. are repeated for all X chromosome sections.

For all B except A: FMF(A) is a set of FMF(A-B); FEC(A) is a set of FEC(A-B).

FEC(A-B) is a value obtained from FEC matrix for a given *Drosophila* strain, being the number of contacts between sections A and B.

5. Specific correlation computation:
  - For each section A: calculation of FEC(A)-FMF(A) Spearman rank correlation coefficient (rho; *p* < 0.05, *n* = 119).
  - Calculation of the average rho value (R) and proportion of statistically significant FEC-FMF cases (P). P is calculated as follows: P = *n* (*p* < 0.05)/total *n* of estimations for which Spearman correlation data were obtained.
6. Unspecific correlation computation: For all different sections A and B: calculation of FEC(A)-FMF(B) Spearman rank correlation rho and *p*. R and P values are calculated as in 5b.
7. Stages 1–6. are repeated at different fragment lengths (L) (10–60 nt, with a step of 5 nt).
8. Excluding DNA microsatellites: Stages 1–7 are repeated with fragment samplings excluding fragments that contain DNA repeats. By default, a repeat contains identical elements in a row: four nucleotides or three dinucleotides or two trinucleotides.
9. Estimation of section proximity effect. By default, the distance (D) between the boundary sections varies from 10 to 116, and only fragments of a specific L (10, 30, 50 nt) are used to compute FEC-FMF correlations. The procedure is performed as follows:
  - For each D: a sequential selection of the X chromosome zones (Z) of D length, with a step of 1 section (e.g., for D = 30, 90 different Z are selected, starting from 1 (Z1, or 1A–5F) and up to 90 (Z90, or 15F–20F)). The section notations A–F are equivalent to 1–6, so Z90 of D30 is also denoted as 156–206.
  - For each Z(D): specific and unspecific R and P calculation, as described in Stages 5–8, taking into account only the sections within Z. Currently, analysis of unspecific correlations is time consuming, taking up about 3 h for each L. So some cases (e.g., with specific L values or excluded repeats) may be omitted to speed up the processes. For each D, R(D) and P(D) values constitute samplings for further statistical analysis. The sampling size *n* is equal to the number of Z(D): *n* = 120–D.

The scheme of the Stages 1–9. is also given in Figure S4.

### 4.3. Statistical Analysis

All analyses were performed using scripts included in the Homology Segment Analysis software package.

- For results obtained at Stages 5–8.: R are compared using a two-sided Mann–Whitney U-test, P are compared using a Chi-square test for two sample proportion comparisons. The parameters of analysis are automatically varied: strain (*CS*/*agn^ts3^*); type of correlation (specific/unspecific); repeats exclusion (“no”/“yes”); chromosome regions (with/without division into sections); type of analysis (comparison of data obtained for different Ls using the same parameters/comparison of data obtained for the same L using different parameters).
- For results obtained at Stage 9.: R and P and compared using a two-sided Mann–Whitney U-test. Samplings obtained for different Ds and Ls are analyzed independently. The parameters of analysis are automatically varied: strain (*CS*/*agn^ts3^*); type of correlation (specific/unspecific); repeats exclusion (“no”/“yes”).

### 4.4. BLAST Analysis of Fragments Contributing to FMF

- For the given L values (here, L30 or L50), the full list of fragments of all sections making contributions to FMF are generated and arranged according to the number of fragment occurrences (NO) > 10, starting from the maximum NO. If the NO is equal for different fragments, only the first fragment is chosen.
- The same procedure is performed for a set of sections showing statistically significant FEC-FMF correlations for the given strain and L value (see Stage 5.).
- The biological nature of the first 50 fragments in each list is revealed using NCBI Blast (http://blast.ncbi.nlm.nih.gov; accessed on 20 February 2021): BLASTN, database—Nucleotide collection, species—*Drosophila melanogaster*, max target sequences—100, other parameters—by default.

## 5. Conclusions

Using our Homologous Segment Analysis software, we have shown a specific positive correlation between FEC and FMF for about 20% of the *CS* X chromosome sections involved in ectopic pairing. Most of the 50 nt fragments specifically contributing to FMF appeared to be related to 372-bp or 1.688 middle repeats. Thus, our bioinformatics approach lets us to handle the problem caused by the low resolution of the method of squashed preparations, which does not give information about the specific sequences involved in ectopic pairing. Using the experimental data on chromatin properties obtained by Hi-C and other 3C-related methods with significantly higher resolutions can substantially increase correlation values and validity. Moreover, Homology Segment Analysis can be easily applied to search correlations between FMF and every feature associated with pairs of genomic regions both in *Drosophila* and in other species. For example, it can be used to find DNA motifs involved in contact formations, as well as the binding of proteins or RNA that mediate such interactions and, thereby, define nuclear 3D organization.

## Data Availability

The FEC data used in this paper are freely available at: https://drive.google.com/drive/folders/1i_SOIR3cxFN1951akkAMGcOgXwd71NG5?usp=sharing (accessed on 12 August 2021).

## Supplementary Materials

The following are available online at https://www.mdpi.com/article/10.3390/ijms22168713/s1.
