# Supplementary material for: Chromatin Structure and “DNA Sequence View”: The Role of Satellite DNA in Ectopic Pairing of the Drosophila X Polytene Chromosome
Source: Int J Mol Sci. 2021 Aug 13;22(16):8713. doi: 10.3390/ijms22168713 (PMC8395981; doi:10.3390/ijms22168713)
Supplement: Supplementary file 1 [file ijms-22-08713-s001.zip › Supplementary materials/Figure S4.pdf]

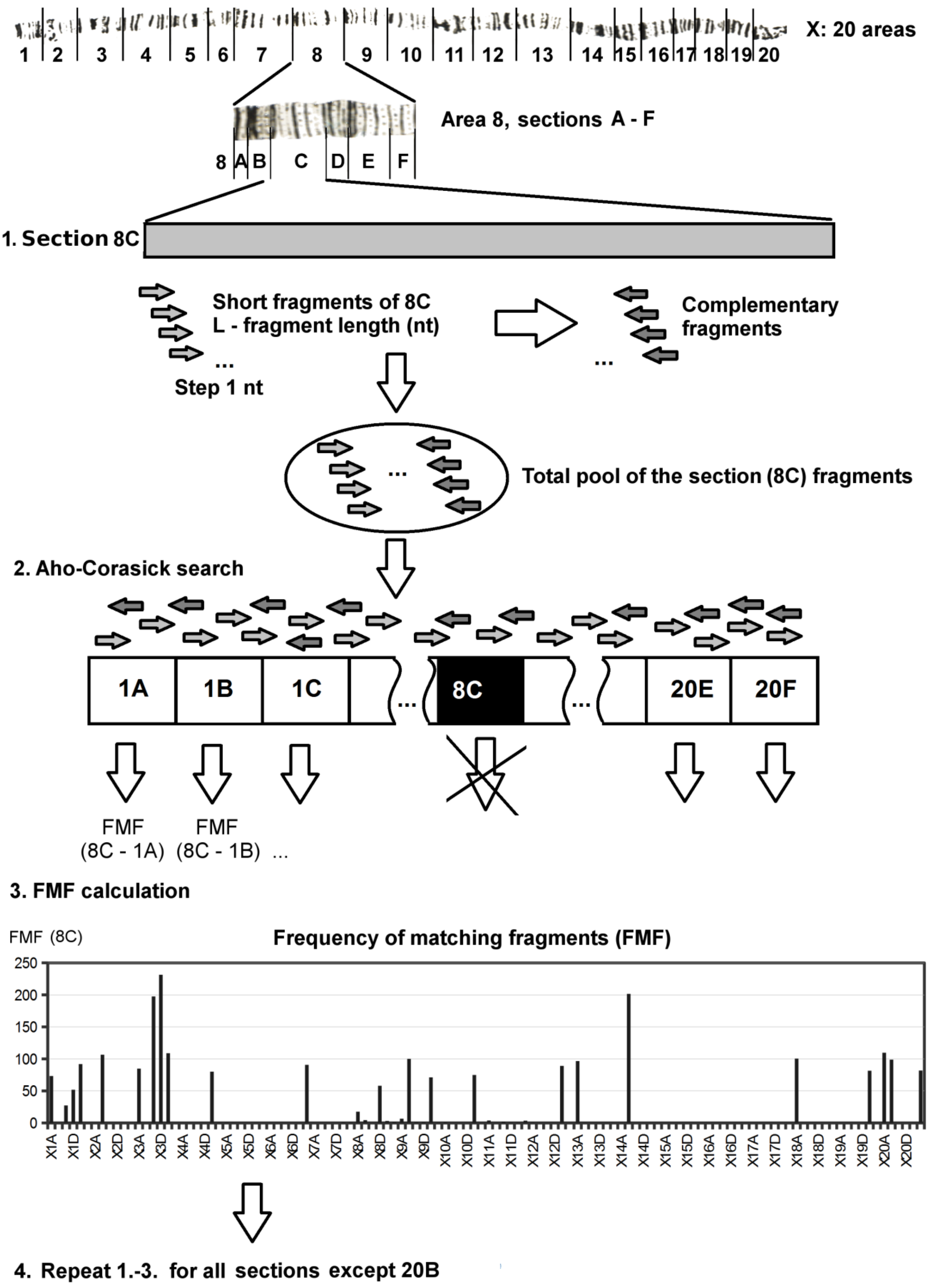

X: 20 areas
1 2 3 4 5 6 7 8 9 10 11 12 13 14 15 16 17 18 19 20
Area 8, sections A - F
8 A B C D E F
1. Section 8C
Short fragments of 8C
L - fragment length (nt)
...
Step 1 nt
Complementary fragments
...
Total pool of the section (8C) fragments
2. Aho-Corasick search
1A 1B 1C ... 8C ... 20E 20F
FMF (8C - 1A)
FMF (8C - 1B) ...
3. FMF calculation
FMF (8C)
Frequency of matching fragments (FMF)
250 200 150 100 50 0
X1A X1D X2A X2D X3A X3D X4A X4D X5A X5D X6A X6D X7A X7D X8A X8D X9A X9D X10A X10D X11A X11D X12A X12D X13A X13D X14A X14D X15A X15D X16A X16D X17A X17D X18A X18D X19A X19D X20A X20D
4. Repeat 1.-3. for all sections except 20B

5. a. FEC - FMF correlation calculation

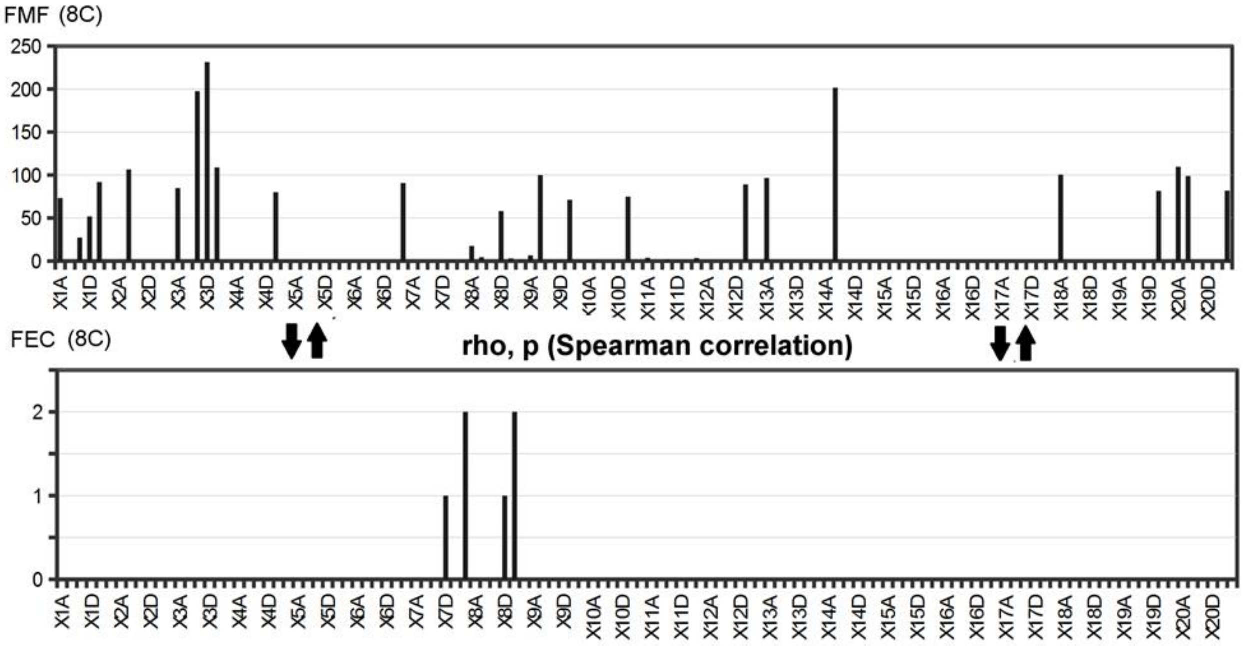

Repeat for all sections except 20B

b. R: the average rho value of significant Spearman correlations ($p < 0.05$)
P: the proportion of significant correlations

6. Unspecific FEC - FMF correlation calculation (for all different sections)

7. Repeat 1. - 6. for all fragment lengths (L)

8. Repeat 1. - 7. for fragments excluding DNA repeats

9. Sections proximity influence on FEC - FMF correlation

a. Zones of D length (D: 10 - 116)

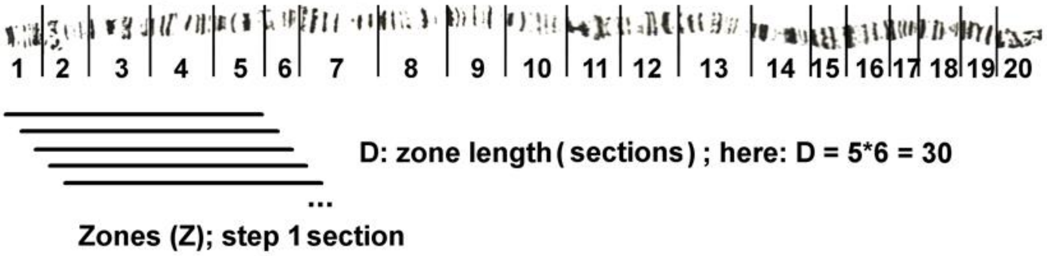

b. Calculate the average values of R and P for all zones of D length

Repeat 9. for all D
