# Supplementary figures and images for: Chromatin Structure and “DNA Sequence View”: The Role of Satellite DNA in Ectopic Pairing of the Drosophila X Polytene Chromosome
Source: Int J Mol Sci. 2021 Aug 13;22(16):8713. doi: 10.3390/ijms22168713 (PMC8395981; doi:10.3390/ijms22168713)

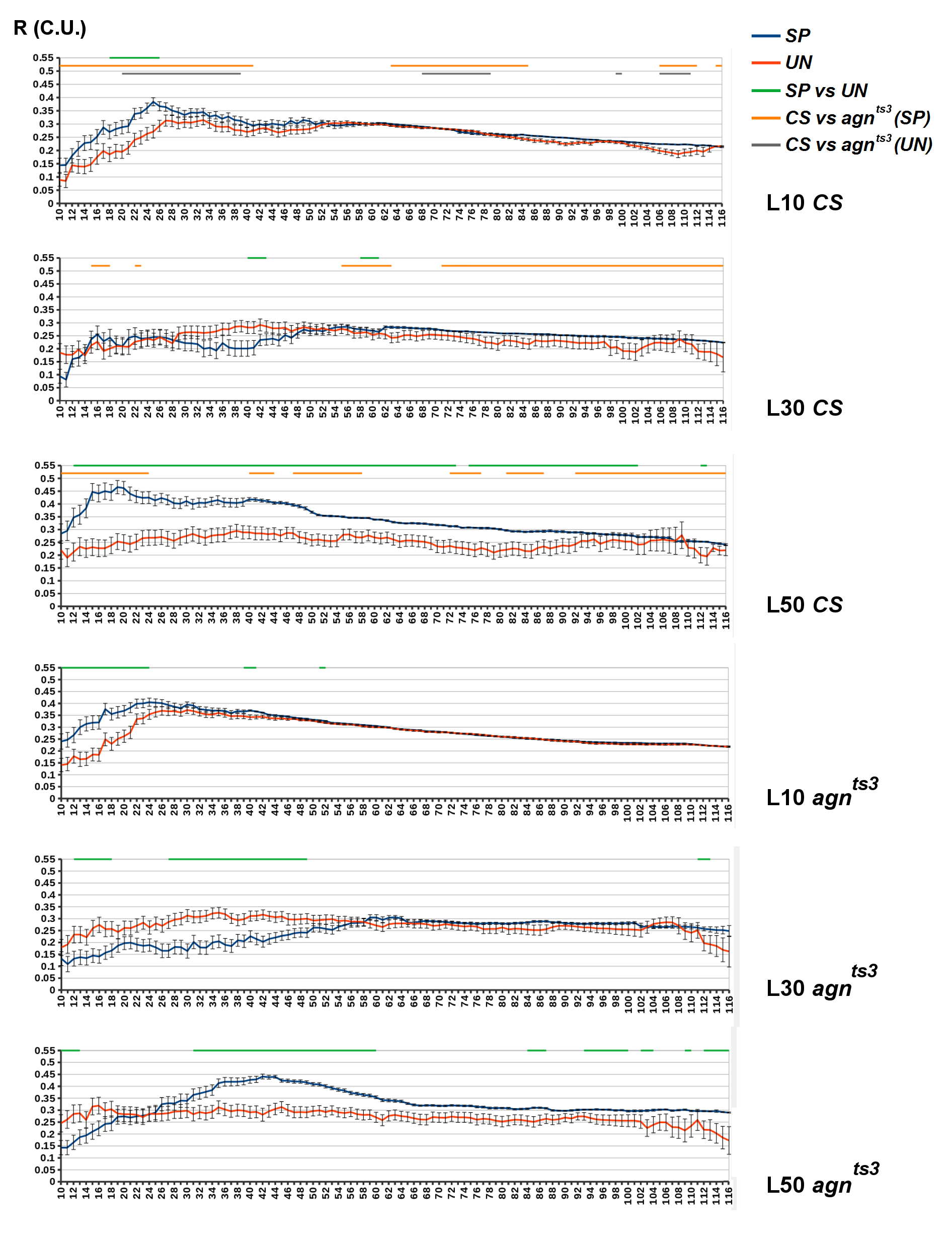

Supplement: Supplementary file 1 [file ijms-22-08713-s001.zip › Supplementary materials/Figure S1.tiff]

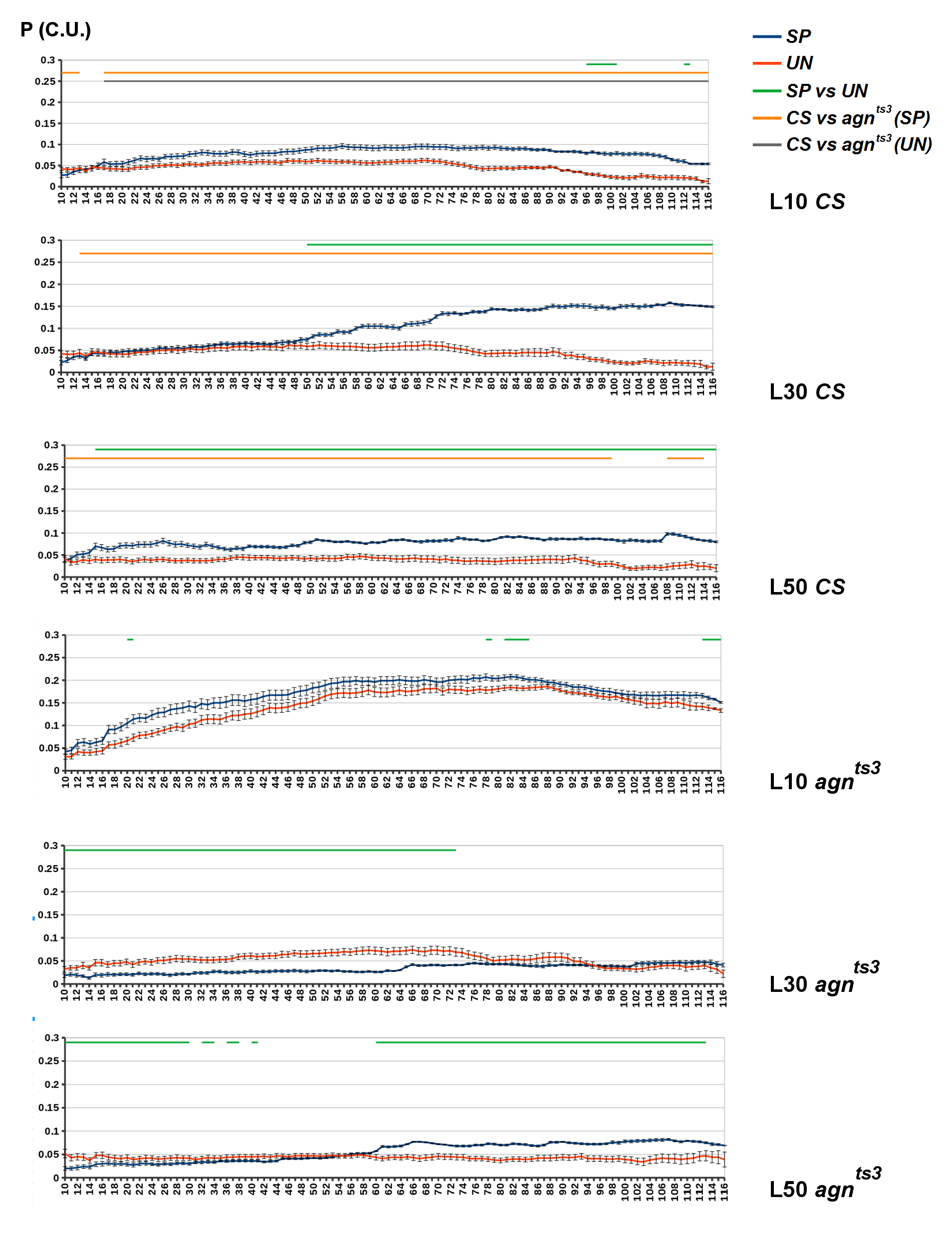

Supplement: Supplementary file 1 [file ijms-22-08713-s001.zip › Supplementary materials/Figure S2.tiff]

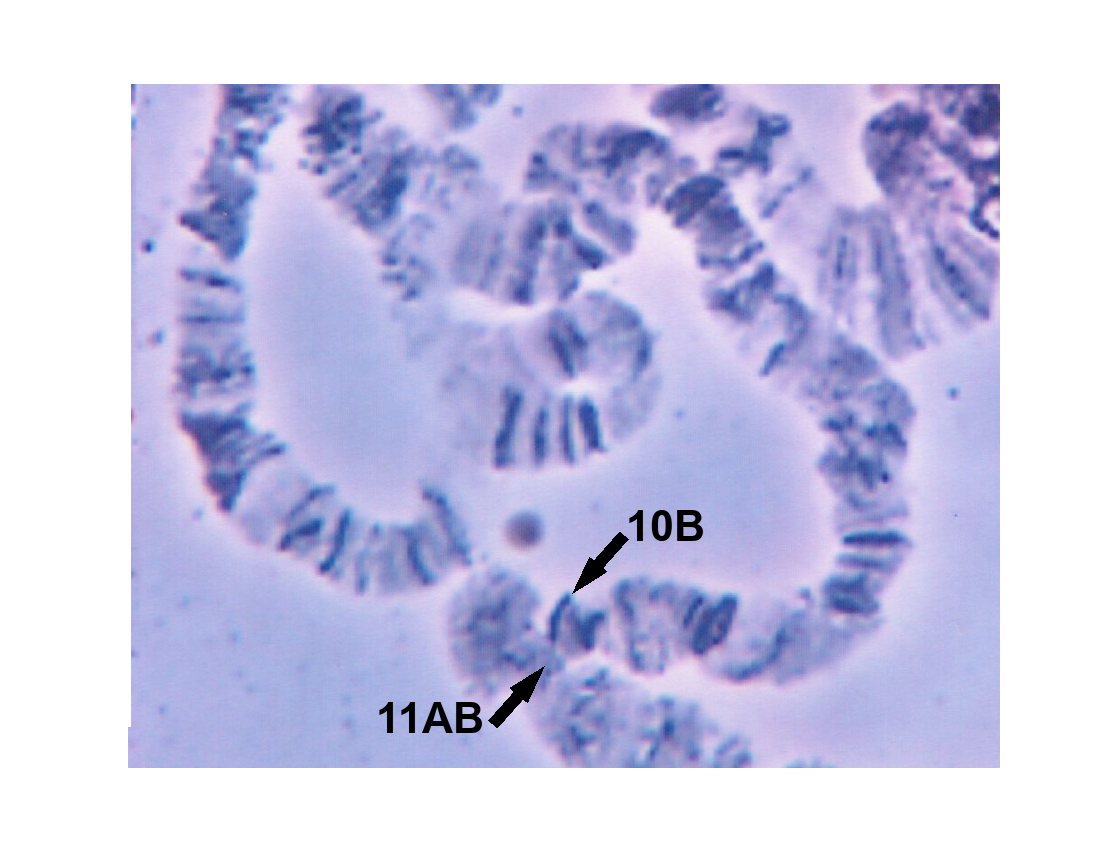

Supplement: Supplementary file 1 [file ijms-22-08713-s001.zip › Supplementary materials/Figure S3.tif]
